# Novel Musculoskeletal Hypotheses in the Armed Services Trauma and Rehabilitation Outcome (ADVANCE) Cohort: Development and Application of Sparse Group Factor Analysis Methodology

**DOI:** 10.2196/91958

**Published:** 2026-07-21

**Authors:** Fraje C E Watson, Fabio S Ferreira, Balasundaram Kadirvelu, Alex N Bennett, Aldo A Faisal, Neil Graham, Harriet Kemp, Paul Cullinan, Christopher Boos, Nicola T Fear, Anthony M J Bull

**Affiliations:** 1 Department of Bioengineering, Imperial College London, 86 Wood Lane, 5th floor, Sir Michael Uren Building, London, England, W12 0BZ, United Kingdom; 2 Department of Computing, Imperial College London, London, England, United Kingdom; 3 Defence Medical Rehabilitation Centre, Loughborough, United Kingdom; 4 Department of Brain Sciences, Imperial College London, London, England, United Kingdom; 5 Department of Surgery and Cancer, Imperial College London, London, England, United Kingdom; 6 Health and Social Sciences, Bournemouth University, Bournemouth, England, United Kingdom; 7 King’s Centre for Military Research and Academic Department of Military Mental Health, King's College London, London, England, United Kingdom

**Keywords:** machine learning, artificial intelligence, traumatic brain injury, musculoskeletal health, military, cohort study, group factor analysis

## Abstract

**Background:**

Musculoskeletal conditions are a leading global cause of disability, yet the factors influencing long-term musculoskeletal health, particularly following trauma, remain incompletely understood. Machine learning could be applied to identify previously unknown patterns in large-scale, multimodal datasets.

**Objective:**

This study aims to test the ability of a new sparse group factor analysis method to uncover hidden patterns in large-scale multimodal datasets and generate testable, clinically relevant hypotheses.

**Methods:**

This study applies sparse group factor analysis, a hierarchical unsupervised machine learning method, to the Armed Services Trauma and Rehabilitation Outcome (ADVANCE) cohort to identify latent structures in multimodal clinical data. ADVANCE is a prospective longitudinal dataset of 1145 UK military personnel and veterans who served in Afghanistan. Half the cohort sustained combat injuries, and the remainder were frequency matched on deployment, service, rank, role, age, and ethnicity. Study 1 validated the approach by rediscovering known group-level patterns between combat-injured and noninjured participants, including poorer outcomes in pain, mobility, and bone health among those with lower limb loss. Study 2 explored the injured, nonamputee subgroup without prespecified labels to identify new hypothesis-generating clusters that could subsequently be tested using standard hypothesis-testing methods.

**Results:**

The ADVANCE cohort was 34.1 (SD 5.4) years old and 8.3 (SD 2.1) years postinjury or 7.7 (SD 1.9) years since matched deployment. A subgroup of 125 individuals with worse musculoskeletal outcomes was uncovered. This group had greater body mass (mean 92.6, SD 14.7 kg vs mean 88.0, SD 13.4 kg; *P*=.002), higher injury severity (median 12, IQR 5-22 vs median 9, IQR 4-14; *P*=.002), and reduced health-related quality of life with head injury. These findings led to a novel hypothesis that head injury, including potential traumatic brain injury, is associated with long-term musculoskeletal deterioration. This hypothesis is supported by literature in both athletic and military populations and will be tested in follow-up analyses.

**Conclusions:**

Our findings demonstrate how sparse group factor analysis, combined with clinical insight, can uncover hidden patterns in large-scale datasets and generate testable, clinically relevant hypotheses that inform prevention, treatment, and rehabilitation strategies.

## Introduction

Musculoskeletal health is an important but complex societal issue. Musculoskeletal disorders are associated with aging [1], genetics [2], occupation [3,4], trauma [5,6], and lifestyle [7] and can be exacerbated by psychosocial factors [8]. Musculoskeletal injury can further compound these challenges [5]. Components associated with increased risk of musculoskeletal injury are multifactorial but include physical fitness, obesity and being underweight [9], age, sex [10], sports such as running [10] and contact sports [11], ergonomics [12], poor neuromuscular control [13], and physically demanding occupations (eg, military) [14,15].

Musculoskeletal conditions are the second-highest cause of global nonfatal disability [16,17]. These conditions affect more than a third of the UK population [18] and 494 million people globally [16]. They result in 2462 disability-adjusted life years per 100,000 globally, which is increasing over time [16,19,20].

A better understanding of the interconnectedness of long-term musculoskeletal outcomes (eg,2 outcomes influence each other, 1 outcome is associated with a particular injury type) associated with injury could guide preventative, interventional, and rehabilitation efforts, potentially decreasing the disability burden and increasing quality life years. Achieving this better understanding would require large, longitudinal datasets. However, looking beyond the expected to generate novel associations or hypotheses in a large dataset is challenging. Artificial intelligence (AI) could provide the necessary processing power and pattern recognition skills required to overcome this challenge. AI has already shown diagnostic, treatment personalization, and prognostic potential in medicine across a number of diagnoses (eg, detecting breast cancer, personalizing sepsis treatment strategies, predicting progression of neurodegenerative diseases) [21-29].

Unsupervised machine learning, a branch of AI, focuses on uncovering hidden patterns and intrinsic structures within unlabeled datasets, without relying on labeled data [30]. Key techniques within unsupervised learning include clustering, dimensionality reduction, and density estimation. These methods are particularly valuable in exploratory data analysis, as they enhance model interpretability and efficiency by elucidating latent structures and reducing complexity. Here, we use sparse group factor analysis (sparse GFA), a hierarchical probabilistic machine learning model to identify subgroup-specific and subgroup-common multimodal latent disease factors in participant groups [31].

The Armed Services Trauma and Rehabilitation Outcome (ADVANCE) study is collecting prospective, longitudinal physical and psychosocial data from a cohort of 1145 UK servicemen who served in the Afghanistan War [32] with the aim of reporting the long-term physical and psychosocial outcomes of combat-related injury. The baseline dataset of ADVANCE contains over 13 million datapoints. Important, predetermined hypotheses that set the recruitment target numbers for the study have been made and tested using musculoskeletal data [6,33-35]. The application of sparse GFA unsupervised machine learning techniques has the potential to uncover hidden patterns in complex datasets to generate previously unseen hypotheses, thus addressing one of the key limitations of analytical techniques currently in use.

This aim of this paper is to describe the methodology undertaken to identify a novel and clinically relevant hypothesis using the musculoskeletal data of the ADVANCE study. To do this, we present two studies: (1) application of sparse GFA to the ADVANCE dataset and comparison of results to known data patterns and (2) application of sparse GFA to a nested study using a subgroup of heterogeneous and underinvestigated ADVANCE participants to generate a novel hypothesis. We hypothesize that GFA can be applied to a complex musculoskeletal dataset and that new clinically relevant hypotheses on causation/correlation can be inferred de novo.

## Methods

### Ethical Considerations

Ethical approval was granted by the UK Ministry of Defence Research Ethics Committee (357/PPE/12). Informed, written consent was obtained from each participant.

### The ADVANCE Cohort

The protocol for the ADVANCE study is described elsewhere [32]. Participants (n=1145) were male serving or veterans of the UK military deployed on combat operations to Afghanistan between 2003 and 2014. Injured participants (n=579, 50.6%) sustained combat injuries requiring aeromedical evacuation to the United Kingdom. Uninjured participants (n=566, 49.4%) were frequency matched by deployment, service, rank, role, and age to injured participants to form a comparison group. The study is limited to men, since fewer than 18 female personnel sustained combat injuries, of whom only 3 had serious or very serious injuries [36].

Baseline data were collected between March 2016 and August 2020, a median of 7.9 (IQR 6.7-9.4) years postinjury or matched deployment.

Comprehensive physical and psychosocial data were collected from all participants across a range of domains, such as cardiovascular, mental health, respiratory health, musculoskeletal health, proteomics, hearing, and aging. This pilot analysis uses the musculoskeletal-adjacent data described in Table 1.

**Table 1.** Type, name, and description of Armed Services Trauma and Rehabilitation Outcome (ADVANCE) variables used to assess ADVANCE study participants and entered into the sparse group factor analysis model.

Method and type	Details
Clinical assessment and self-reported	
Demographic	Age, height, (adjusted) body mass, NISS^a^, socioeconomic status, BMI, abdominal circumference, waist-to-hip ratio, race, service, role, rank, smoking status, amputation status
DEXA^b^ of the whole body, right and left proximal femur, and lumbar spine	
Bone mineral density	t-score for the left and right hip (femoral neck, trochanter, Ward’s triangle, intertrochanter, hip total), t-score for the L1-L4 spine, raw bone mineral density for each lumbar vertebra (L1-L4), bone mineral density for the whole body
Body composition	Total lean tissue in the whole body, percentage of fat tissue in the whole body
Posterior-anterior semiflexed knee radiographs	
Radiographic knee osteoarthritis	Kellgren-Lawrence score (0‐4) [37], OARSI^c^ joint space narrowing score (0‐3), OARSI osteophyte score (0‐3), OARSI sclerosis score (0‐3) [38], minimum medial joint space width, and minimum lateral joint space width for left and right knees
Physical assessment—all participants	
Six-minute walk test	Furthest distance a participant can walk in 6 minutes
Physical assessment—lower limb loss only	
Amputee Mobility Predictor [39]	Ability to perform 21 tasks scored by independent assessor scored 0‐47, where a higher score is indicative of better mobility
Questionnaire—all participants	
ASAS^d^ definition of inflammatory back pain [40]	Five questions about back pain to identify inflammatory back pain (suspected when ≥4 answers are yes)
Health-related quality of life score (EQ-5D-5L^e^) [41]	Health-related quality of life score for individual items; mobility, self-care, usual activities, pain or discomfort, anxiety or depression each scored 1‐5, where a higher score is a worse outcome, and overall health scored 0‐100, where 100 is the best health.
Back pain	Scored for severity, frequency, and impact 0‐10, where 10 is the severe, frequent, or impactful.
ODI^f^ [42]	Ten-item questionnaire each scored 0‐5 with a total score of 0‐100, where 100 is indicative of the most back pain–related disability.
KOOS^g^—pain [43]	Nine-item questionnaire each scored 0‐5 for left and right knees, each with a total score of 0‐100, where 0 is indicative of the worst pain.
KOOS—symptoms [43]	Seven-item questionnaire each scored 0‐5 for left and right knees, each with a total score of 0‐100, where 0 is indicative of the worst symptoms.
Knee and Hip Joint Pain scores	Left and right knees and hips scored for severity, frequency, and impact 0‐10, where 10 is the severe, frequent, or impactful
NAHS^h^	Twenty-item questionnaire each scored 0‐5 for left and right hips, each with a total score of 0‐100 for each hip, where 0 is indicative of the worst hip pain, symptoms, activities, or function.
DASH^i^—core [44]	Thirty-item questionnaire each scored 0‐5 with a total score of 0‐100, where 100 is indicative of the most disability
DASH—work [44]	Four-item questionnaire each scored 0‐5 with a total score of 0‐100, where 100 is indicative of the most disability
DASH—sport [44]	Four-item questionnaire each scored 0‐5 with a total score of 0‐100, where 100 is indicative of the most disability
IPAQ^j^ [45]	Total weekly activities categorized as moderate (“activity that makes you breathe somewhat harder than before”) and vigorous (“activities that take hard physical effort and make you breathe much harder than normal”)
Questionnaire—lower limb loss specific	
SIGAM^k^ Mobility Questionnaire [46]	Scored A-F based on self-reported mobility
Socket comfort score [47]	Scored per amputated limb 0‐10, where 10 is the most comfortable
Prosthetic satisfaction score [47]	Scored per amputated limb 0‐10, where 10 is the most satisfaction
Phantom pain score	Scored for severity, frequency, and impact 0‐10, where 10 is the severe/frequent/impactful
Stump pain score	Scored for severity, frequency, and impact 0‐10, where 10 is the severe, frequent, or impactful

a NISS: New Injury Severity Score.

b DEXA: dual X-ray absorptiometry.

c OARSI: Osteoarthritis Research Society International.

d ASAS: Assessment of SpondyloArthritis International Society.

e EQ-5D-5L: EuroQoL 5-Dimensions 5-Level.

f ODI: Oswestry Disability Index.

g KOOS: Knee Osteoarthritis Outcome Score.

h NAHS: Non-Arthritic Hip Score.

i DASH: Disability of the Arm, Shoulder and Hand.

j IPAQ: International Physical Activity Questionnaire.

k SIGAM: Special Interest Group in Amputee Medicine.

For participants with limb loss, adjusted body mass was calculated depending on body segments missing [48,49] and without wearing prosthetics. The height of participants with lower limb loss was measured using either actual or reported height.

Injuries sustained by the injured group were identified using the Joint Theatre Trauma Registry (JTTR), which details combat injury type and location.

### Data Handling

#### Data Processing

Data were preprocessed with a bespoke Python workflow. For the nested study addressing the second aim, this workflow was applied exclusively to the injured cohort excluding those with lower limb loss (n=422). Focusing on this subcohort allowed the assessment of heterogeneity in musculoskeletal outcomes without the confounding influence of limb loss.

All 422 participants were retained through preprocessing. Some records contained item-level missingness before imputation, which affected 231 questionnaire records, 35 anthropometric records, 12 x-ray records, 9 blood records, 7 left hip dual X-ray absorptiometry (DEXA), 5 right hip DEXA, and 3 spinal DEXA records, and 2 pain-location records, in most cases involving only a small number of items per record. Missingness was addressed using joint multivariate iterative imputation fitted over the concatenated multimodal feature matrix (scikit-learn IterativeImputer with a decision-tree base learner, 100 iterations), allowing missing values in 1 modality to be informed by observed values in the others. We assumed missingness was conditionally random, as it was driven primarily by logistical factors rather than underlying clinical constructs. We did not perform complete-case exclusion, and the final dataset retained all 422 participants.

#### Cohort Curation

Participants labeled (injured, nonamputee) in the study metadata were retained. Questionnaire variables were kept at the total- or domain-score level to minimize multicollinearity and reduce the risk of model overfitting; individual item scores were discarded. All musculoskeletal-adjacent variables listed in Table 1 were imported and subjected to range and type checks. Records with missing or implausible values in key demographic or musculoskeletal fields (eg, body mass, New Injury Severity Score [NISS]) were removed.

#### Modality Construction

Features were organized into separate modality-specific matrices for self-reported outcomes, clinical assessments, and imaging-derived measures, consistent with the sparse GFA framework. Continuous variables were standardized to zero mean and unit variance, and categorical variables were one-hot encoded. Outliers were retained because the hierarchical Bayesian formulation of sparse GFA is robust to extreme observations, and such extremes are clinically informative in trauma cohorts.

To ensure comparable latent-factor estimation across modalities, only participants with complete data for every retained modality were included in the final analysis set. The resulting multimodal matrix constituted the input to the sparse GFA model described in the subsequent section and represents a harmonized, quality-controlled view of musculoskeletal health in the injured nonamputee group.

### Data Analysis

#### Sparse GFA

Sparse GFA identifies latent factors in subgroups that capture the relationships within multimodal data [31]. Sparse GFA is an extension of GFA [50], where regularized horseshoe priors are added over the loading matrices and latent variables. The regularized horseshoe prior [51] is a popular shrinkage prior used in sparse Bayesian regression to ensure that small coefficients are heavily shrunk toward zero, while large coefficients are regularized but remain large. In this way, sparse GFA improves model interpretability and identifies latent variables that are differently expressed across the sample.

In this study, sparse GFA was fitted using a Markov chain Monte Carlo algorithm with 5 sampling chains and 4000 samples (the first 1000 were discarded as warm-up). The inference was randomly initialized 5 times, and the best initialization was selected to maximize the expected log joint posterior density. All sampling chains were initialized with 20 factors. More details about the experiment setup are given in Ferreira et al [31].

We used the approach proposed by Ferreira et al [31] to select robust factors, that is, the factors are first averaged over the posterior samples within a sampling chain and then compared to the factors obtained in other sampling chains using cosine similarity. Two factors were considered the same if the cosine similarity between them was greater than 0.80, as previously described [52]. A factor was considered robust if it had been obtained in at least half of the sampling chains.

#### Interpretation

For each participant, sparse GFA calculates a distribution across the latent variables, showing how a particular participant is represented in each component. For each component, we also obtain a representation across the features in each data modality. This provides a loading matrix showing the importance of each feature in each component for each participant, which we refer to as *data components*. These data components allow the interpretation of the results (eg, a positive [more red] value for a particular participant can be interpreted as scoring high in a task).

**Figure 1.**
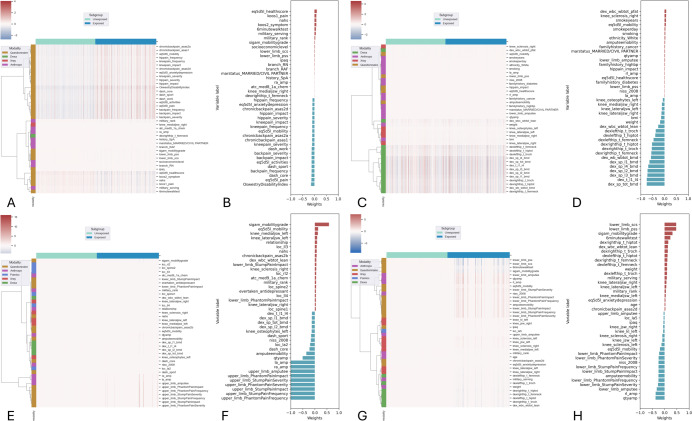
Study 1—top 4 data components identified by sparse group factor analysis in the uninjured and injured participants in the Armed Services Trauma and Rehabilitation Outcome (ADVANCE) dataset and their associated weights (A and B, C and D, E and F, and G and H). Blue represents a value below the sample average, and red represents a value above the sample average. Colors on the left identify up to 5 different modalities or types of data, as listed for each factor.

In study 1, data components were considered where there was a clear difference between the injured and uninjured groups (eg, Oswestry Disability Index scores were largely red for the injured group and blue for the uninjured group; Figure 1A). The hypothesis regarding this difference was inherent due to the known way the data were split into groups. The injured group contained a subgroup of 157 men who sustained injuries resulting in limb loss. They were not labeled but could be identified through limb loss-specific outcomes (eg, phantom limb pain), or where nonspecific outcomes (eg, low bone mineral density) were shown in combination with limb loss-specific measures.

In study 2, a nested subset of injured participants without lower limb loss injuries was analyzed without an inherent group structure to demonstrate a real-world application of sparse GFA that could potentially generate de novo hypotheses on causation or correlation. Loading matrices are analyzed to identify a group structure (eg, subgroup has poor scores across specific outcomes). Next, patterns in the data were investigated and used to develop a novel hypothesis. In this example, the following steps were taken:

1. Group structure identified a subgroup with poor scores across specific outcomes.
2. Participants were manually divided into those with and without poor scores for those specific outcomes.
3. Injury location, demographic information, and injury severity were visually and statistically compared between groups.
4. Overall health EuroQoL 5-Dimensions 5-Level (EQ-5D-5L) scores were visually compared between injury location groups.

#### Statistical Analysis

The JTTR records were unavailable for 41 injured participants who were subsequently excluded from analyses involving injury location or NISS (higher score=greater severity).

In the first study, 1 uninjured participant was excluded due to noncombat-related lower limb loss following matching.

In the second study, where statistical comparisons of continuous variables were made between the groups, the data were visually assessed for normality using histograms, and an independent 2-tailed *t* test, Mann-Whitney *U* test, or chi-squared test was used, as appropriate. Boxplots were compared visually for trends. Descriptive data and statistical analysis were processed using Stata SE version 18.5.

## Results

### Demographic Information

Across the entire cohort, the mean age was 34.1 (SD 5.4) years, the mean height was 179.1 (SD 6.8) cm, and the mean (adjusted) body mass was 89.0 (SD 13.3) kg. Participants self-described their race as White (n=1036, 90.6%), Black (n=46, 4.0%), Asian (n=41, 3.6%), or Mixed/Other (n=21, 1.8%). Data were collected from injured participants (those who sustained combat injuries in the Afghanistan war requiring aeromedical evacuation to the United Kingdom) at a mean of 8.3 (SD 2.1) years postinjury and from uninjured participants (frequency-matched participants who did not sustain combat injuries in the Afghanistan war requiring aeromedical evacuation to the United Kingdom) at a mean of 7.7 (SD 1.9) years since matched deployment.

### Sparse Group Factor Analysis

#### Study 1

Fourteen data components were identified (Supplementary Material S1 in Multimedia Appendix 1), of which the top 4 are shown in Figure 1. Table 2 shows the dominant variables that were different between injured and uninjured groups within each factor and overall interpretation.

**Table 2.** Data components identified in study 1 analysis, which can be seen in the raw data output in Figure 1.

Group and type	Outcome variable
Injured	
Back pain	More symptoms associated with spondyloarthritis (Figure 1A) Worse severity, frequency, and impact of low back pain (Figure 1A) Worse functional disability (ODI^a^ score) (Figure 1A)
Health-related quality of life	Worse EQ-5D-5L^b^ scores for mobility, depression and anxiety, pain, and ability to carry out their usual activities (Figure 1A) Worse overall health score (Figure 1A) Worse 6MWT^c^ distance (Figure 1A)
Knee pain	Worse frequency, severity, and impact of knee pain (Figure 1A) Worse KOOS^d^ pain and KOOS symptoms scores (Figure 1A)
Hip pain	Worse frequency, severity, and impact of hip pain (Figure 1A) Worse NAHS^e^ scores (Figure 1A)
Upper limb function	Worse DASH^f^ scores (Figure 1A)
Lifestyle	Less often still serving in the military (Figure 1A)
Injured—lower limb loss	
Amputee-specific	Phantom limb pain (frequency, severity, impact; Figure 1G) Residuum pain (frequency, severity, impact; Figure 1G) Poor SIGAM^g^ grade (Figure 1G)
Mobility	Poor EQ-5D-5L mobility scores (Figure 1G) Short 6MWT distances (Figure 1G)
Knee radiographs	High Kellgren-Lawrence score (Figure 1G) High score for sclerosis (Figure 1G) High score for medial joint space narrowing (Figure 1G)
Bone mineral density	Low bone mineral density in hip (Figure 1G)
Demographic	High injury severity (NISS^h^) (Figure 1G)

a ODI: Oswestry Disability Index.

b EQ-5D-5L: EuroQoL 5-Dimensions 5-Level.

c 6MWT: Six-Minute Walk Test.

d KOOS: Knee Injury and Osteoarthritis Outcome Score.

e NAHS: Non-Arthritic Hip Score.

f DASH: Disability of the Arm, Shoulder and Hand.

g SIGAM: Special Interest Group in Amputee Medicine.

h NISS: New Injury Severity Score.

In Figure 1C,D, bone mineral density was identified as a key component, but it was not explained by a difference between the injured and uninjured groups.

Many of these clusters have already been investigated using hypothesis-driven statistical analyses by the ADVANCE study. For example, we have previously reported higher knee and hip Kellgren-Lawrence, Disability of the Arm, Shoulder and Hand, Oswestry Disability Index, overall pain, and back pain scores in the injured group [6,34,53-55], and participants with lower limb loss have significantly lower bone mineral density in their amputation-side femoral neck [33].

#### Study 2

##### Overview

**Figure 2.**
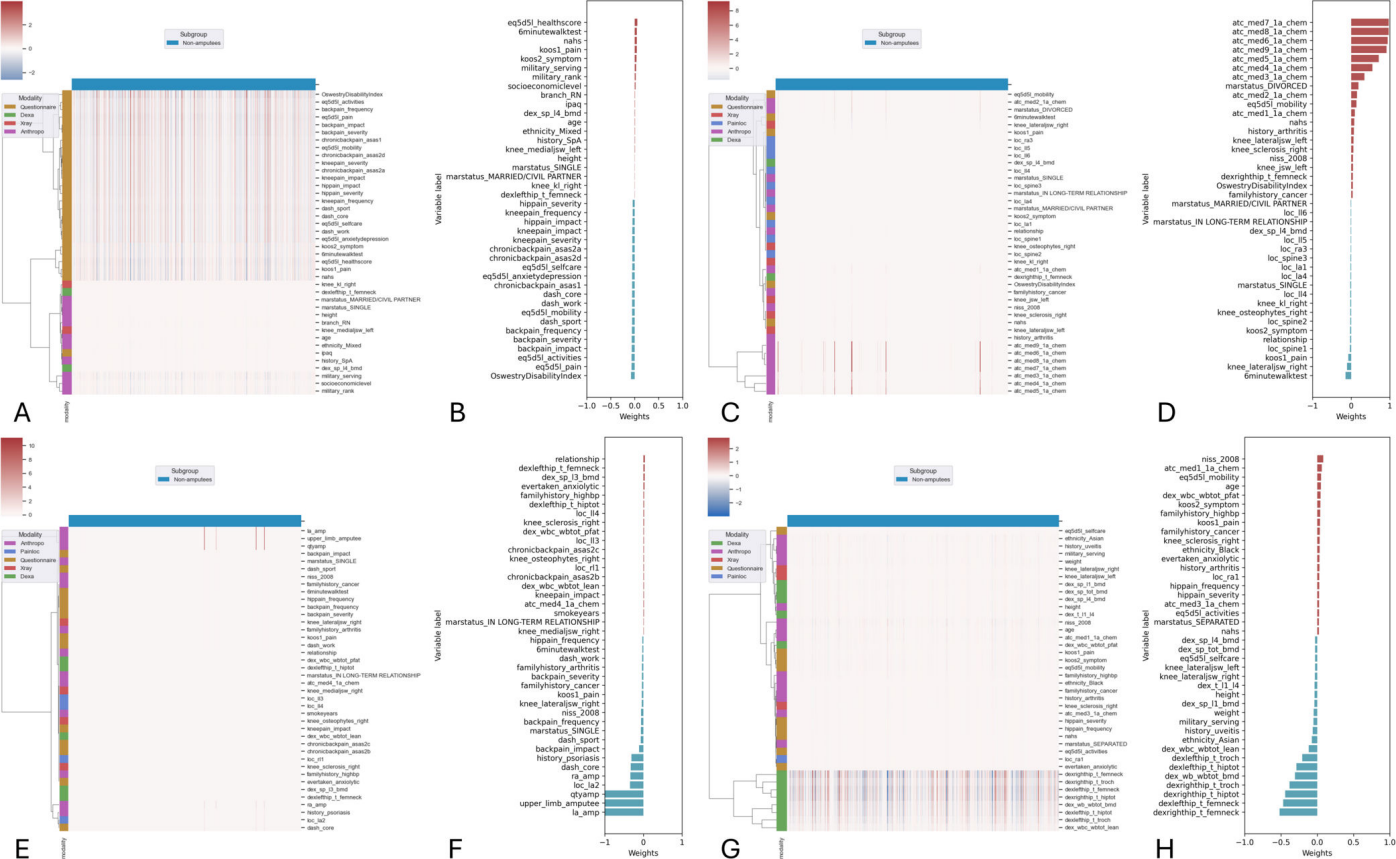
Study 2—top 4 data components identified by sparse group factor analysis in the injured, nonamputee participants in the Armed Services Trauma and Rehabilitation Outcome (ADVANCE) dataset and their associated weights (A and B, C and D, E and F, and G and H). Blue represents a value below the sample average, and red represents a value above the sample average. Colors on the left identify up to 5 different modalities or types of data, as listed for each factor.

Fourteen data components were identified (Supplementary Material S2 in Multimedia Appendix 1), of which the top 4 are shown in Figure 2.

Following the steps described above, a group structure was identified in Figure 2A, which identified a group with high (red) scores across multiple outcomes and low (blue) scores for some other outcomes (Table 3). The threshold for a variable being red and blue is the sample mean. Next, participants were split into 2 groups along the identified structure where the change from red to blue or blue to red occurred across participants. In this instance, this led to a group of 125 participants with poor musculoskeletal outcomes, and the remaining 297 participants without them. Finally, patterns in the demographic, injury pattern, and overall health outcome data were analyzed to identify possible reasons for worse musculoskeletal outcomes.

**Table 3.** Data components identified in round 2 analysis.

Group and type	Outcome variable
Subgroup of 125	
Back pain	More symptoms associated with spondyloarthritis (Figure 2A) Worse severity, frequency, and impact of low back pain (Figure 2A) Worse functional disability (Figure 2A)
Health-related quality of life	Worse EQ-5D-5L^a^ scores for mobility, depression and anxiety, pain, and ability to care for themselves (Figure 2A) Worse overall health score (Figure 2A) Worse 6MWT^b^ distance (Figure 2A)
Knee pain	Worse frequency, severity, and impact of knee pain (Figure 2A) Worse KOOS^c^ pain and KOOS symptoms scores (Figure 2A)
Hip pain	Worse frequency, severity, and impact of hip pain (Figure 2A) Worse NAHS^d^ scores (Figure 2A)
Upper limb function	Worse DASH^e^ scores (Figure 2A)
Demographic	Less often still serving in the military (Figure 2A) Lower military rank (Figure 2A)

a EQ-5D-5L: EuroQoL 5-Dimensions 5-Level.

b 6MWT: Six-Minute Walk Test.

c KOOS: Knee Injury and Osteoarthritis Outcome Score.

d NAHS: Non-Arthritic Hip Score.

e DASH: Disability of the Arm, Shoulder and Hand.

##### Pattern Identification

There were no significant differences in age (mean 34.5, SD 5.7 y vs 34.2, SD 5.2 y), height (mean 179.1, SD 6.9 cm vs 178.6, SD 6.2 cm), or time since injury (median: 8.5, IQR 7.1-10.0 y vs 8.7, IQR 7.0-10.3 y) between the groups with and without poor musculoskeletal outcomes. The group with poor musculoskeletal outcomes was heavier (mean 92.6, SD 14.7 kg) and had higher NISS scores (median 12, IQR 5‐22) than those without (mean weight 88.0, SD 13.4 kg; median NISS 9, IQR 4‐14); both comparisons were statistically significant (*P*=.002).

**Figure 3.**
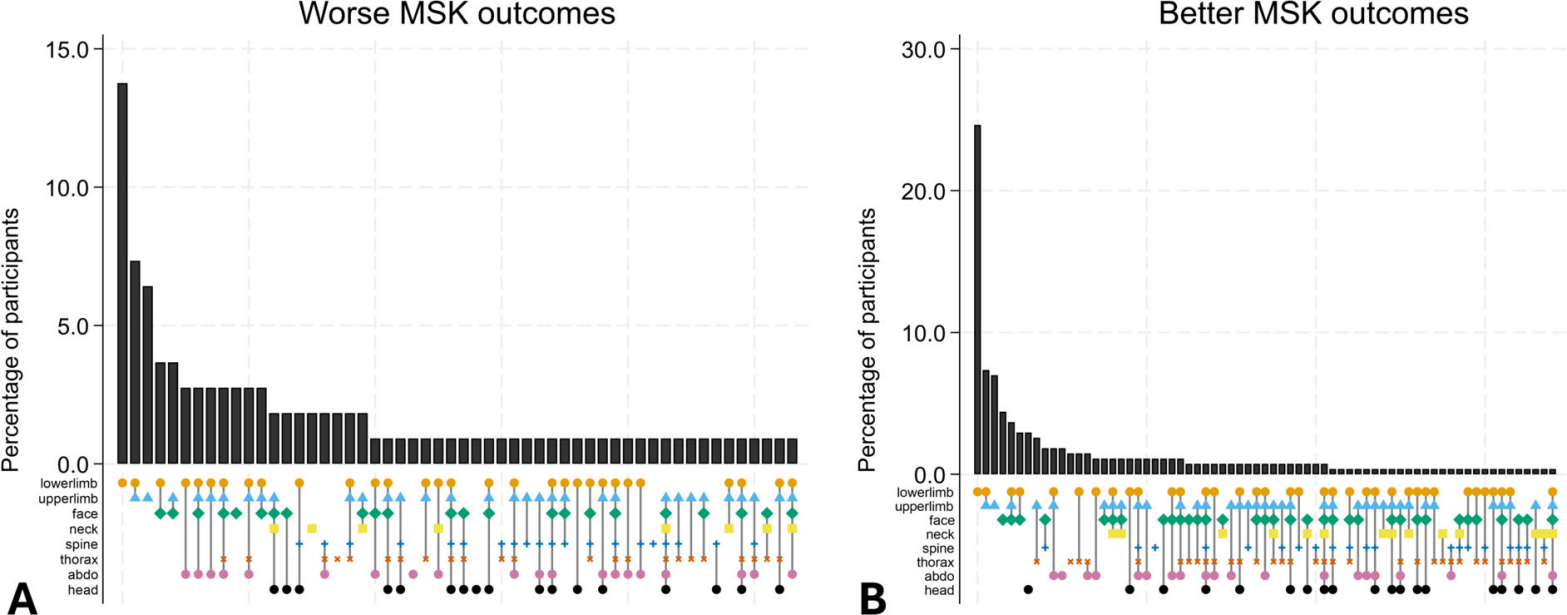
UpSet plot showing injury location combinations for participants in the injured, nonamputee cohort with (A) and without (B) poor musculoskeletal (MSK) outcomes.

The group with poor musculoskeletal outcomes contained a significantly higher percentage of participants with upper limb (n=61, 56.0% vs n=116, 42.7%; *P*=.02), abdominal (n=31, 28.4% vs n=46, 16.9%; *P*=.01), and spinal injuries (n=23, 21.1% vs n=33, 12.1%; *P*=.03) compared to the group with better musculoskeletal outcomes. For injury combination, both groups had the same top three: lower extremity injuries only (n=15, 13.8% and n=67, 24.6%), a combination of upper and lower extremity injuries (n=8, 7.3% and n=20, 7.4%), and upper extremity injuries only (n=7, 6.4% and n=19, 7.0%; Figure 3).

**Figure 4.**
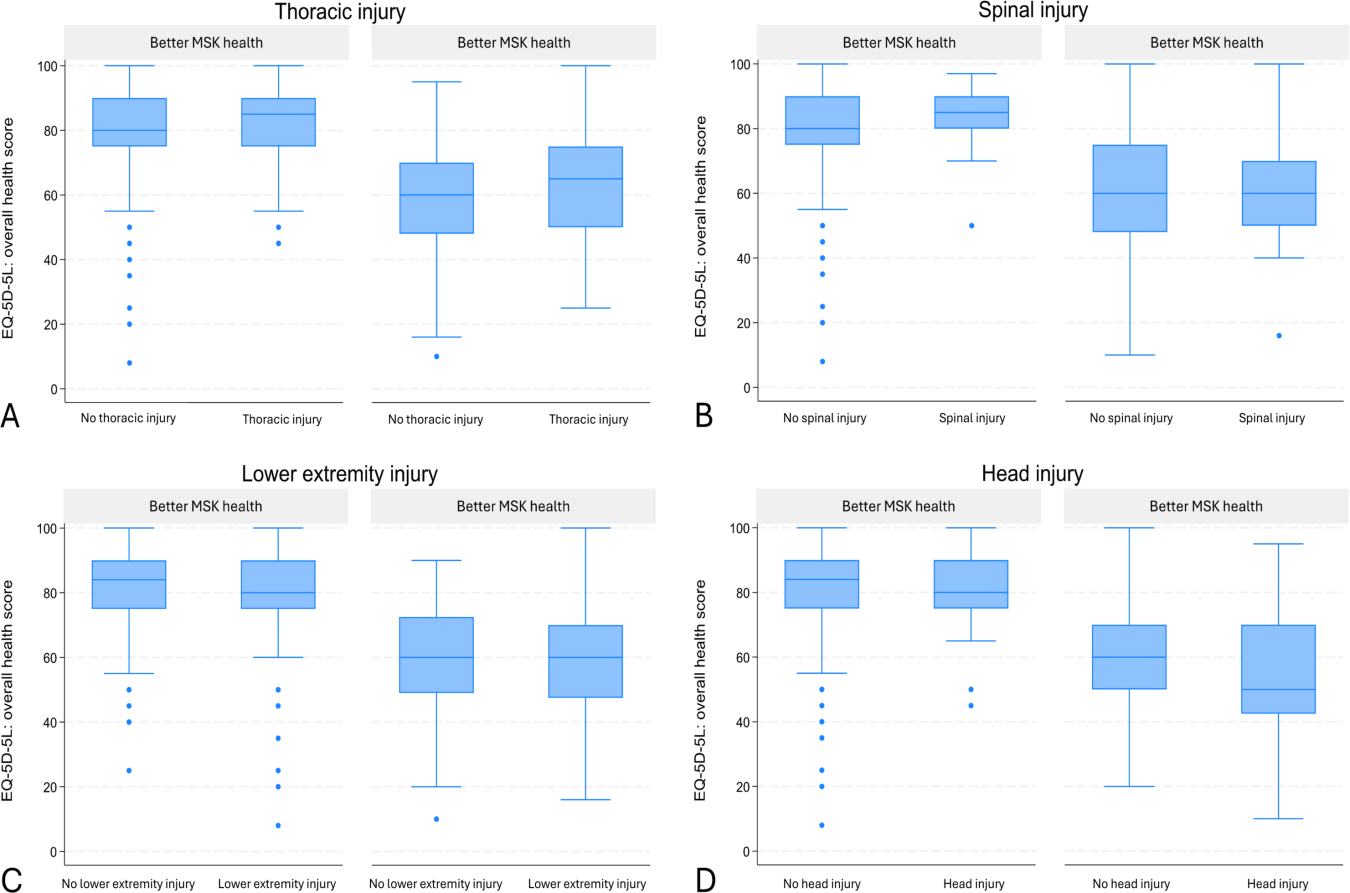
Boxplot of overall health score for injured, nonamputee participants with and without poor musculoskeletal (MSK) outcomes, by presence/absence of a thoracic (A), spinal (B), lower extremity (C), or head (D) injury.

The visual inspection of boxplots further split each group by the presence or absence of an injury in each location and compared overall health score. As expected, scores for those with poor musculoskeletal outcomes were always worse; however, head injury (Figure 4D) was the only body region to worsen the overall health score further in the group with poor musculoskeletal outcomes. Full results are provided in Supplementary Material S2 in Multimedia Appendix 1.

Pattern identification revealed the following hypotheses regarding worse musculoskeletal outcomes in a subgroup of the injured, nonamputees: greater body mass, worse injury severity, and head injury.

## Discussion

### Principal Findings

This work has demonstrated a method for generating novel hypotheses in multidimensional data using sparse GFA. In study 1, we showed how sparse GFA was able to identify known clusters in the cohort, thus corroborating their value. Study 2 showed how sparse GFA was able to identify a cluster with particularly poor musculoskeletal outcomes in a cohort with a heterogeneous internal structure. We investigated patterns in the cluster to generate hypotheses about the potential cause. Specifically, in a cluster of participants with worse musculoskeletal outcomes, we identified higher injury severity score, higher body mass, and head injury as potential causes. The novel head injury hypothesis could be investigated further using typical statistical methods.

In study 1 using the full ADVANCE cohort, we were able to identify patterns that had already been addressed, thus confirming the validity and usefulness of the model. Many of these findings were heavily influenced by scores from the participants with lower limb loss, consistent with the ADVANCE study’s research, where their musculoskeletal outcomes are often worse [6,33-35,53-55]. Sparse GFA also generated hypotheses on hip pain across the injured and uninjured groups that were already being investigated when this study was performed. Study 1 identified a pattern in bone mineral density findings that was not explained by injured or uninjured status. Future consideration of that pattern, such as that demonstrated in study 2, could be applied here to formulate a hypothesis.

Having confirmed the validity of sparse GFA, we subsequently analyzed the injured-, nonamputee group separately, as they are a more heterogeneous group without a clear internal structure that has not been investigated. Using one loading matrix as an example, sparse GFA identified a group of 125 participants with similarly poor musculoskeletal outcomes. The exploration of patterns delineating these groups found that those with worse musculoskeletal outcomes were significantly heavier, which is logical as obesity is often linked to worse musculoskeletal outcomes [56]. The group with worse musculoskeletal outcomes had significantly worse injury severity scores, which also explains worse outcomes. Using injury and overall health outcome data, we identified head injury as a possible correlate of worse musculoskeletal outcomes. This methodology has identified a new hypothesis for the ADVANCE study; long-term musculoskeletal outcomes of participants who sustained a head injury are worse than participants who did not. However, this hypothesis requires independent validation before any inferential claims can be made.

A head injury could lead to a traumatic brain injury (TBI) either as a result of direct impact to the head or secondary to a blast [57]. A review of the literature on head injuries, TBI, or concussion and musculoskeletal injury established that this is a developing field in professional athletes and with existing reports on military personnel. A systematic review and meta-analysis of 8 studies found 2 times higher odds of musculoskeletal injury in athletes following a concussion up to 2 years later [58], with similar findings in a meta-analysis of 27 papers including athletes and military personnel [59]. Military personnel who sustained a concussion (ie, a mild TBI) were 1.84 times more likely to sustain an upper limb musculoskeletal injury in the subsequent year [60]. A long-term outcome study in people with a moderate-to-severe TBI more than 15 years prior reported high rates of musculoskeletal complaints [61]. There is no consensus on the neuropathology of these findings, but some studies suggest impaired neuromuscular control [62] and difficulty with dual-task control [63] following TBI.

Based on the findings from this study, the association between head or facial injury, TBI, and poor musculoskeletal outcomes in military personnel will be investigated in the ADVANCE cohort using hypothesis-driven statistical methods. In the ADVANCE study, incidence of index and lifetime TBI has been defined using the Ohio State University TBI Identification Method, including the clinical assessment of brain magnetic resonance imaging. The novel hypothesis could be tested within a subset of participants with no lifetime TBI and only an index TBI at the point of injury to strengthen the conclusions made about TBI. Regression models adjusted for confounders could compare musculoskeletal scores between groups. Once available for at least 3 timepoints, longitudinal data will be compared over time using a mixed effects ordinal regression model. The density of prospective, longitudinal musculoskeletal outcome data with longer follow-up times than most existing literature will ensure a unique contribution to the literature. These findings will inform guidance on the treatment of TBI, long-term outcomes, and identification of areas for rehabilitation.

Sparse GFA could be applied to a larger, more complex dataset across multiple health care–related modalities such as mental health, cardiovascular health, proteomics, and brain magnetic resonance imaging. Many of the clusters and hypotheses formed in this pilot study had already been addressed because it is likely that different musculoskeletal outcomes will be related to each other. Introducing more data types across specialties could uncover a previously unseen or unknown interconnectedness between data themes. This method blends the high processing power of AI while retaining a rational clinical input to generate novel hypotheses that can be further tested using traditional statistical techniques.

### Limitations

This study identified injury locations using the JTTR, which has limitations as the information is documented in a live war zone and active trauma care setting, which could result in inaccuracies. There is potential that injuries are missed and not documented at this stage, such as head injuries, which could affect the robustness of the hypothesis. Additionally, other injuries could contribute to TBI, not just head injuries, as well as the influence of the NISS. To overcome this, future testing of this new hypothesis will incorporate a thorough and robust analysis of potential TBI to more accurately define head injury status, adjusting for NISS and other potential confounders as required.

The JTTR defines head and face injuries separately, so only head injuries contribute to this hypothesis, but some facial injuries are also linked to TBI [64]. The ADVANCE study is currently working on defining TBI using information from the JTTR, self-reported past medical history, and the Ohio State Questionnaire, with severity defined using the Mayo Classification System. This classification will allow for a comprehensive assessment of TBI and a thorough investigation of the hypothesis.

This study uses data from a male, military cohort, which challenges the direct generalizability of the novel hypothesis it generated to the general population and/or female cohorts. However, presented here is a methodology that could be used in the general population (eg, using UK Biobank data) and/or female military cohorts in future research. The specific novel hypothesis described here could be generalized to male civilians who have been exposed to combat-related trauma, a circumstance that is sadly increasing worldwide. The “overall health today” outcome of the EQ-5D-5L was used to test the effect of injury location as it was not body region specific. Doubts have been cast on the EQ-5D-5L, which has been considered to have a ceiling effect and to be insensitive to change in some populations, despite its strong psychometric properties [65]. Finally, the use of ADVANCE musculoskeletal data only could have biased the results compared to using the full ADVANCE dataset, but this is inherent to the pilot nature of this work and we plan to apply it to the full dataset in the future.

### Conclusions

This application of sparse GFA combined with human interpretation can be used to identify hidden patterns and intrinsic structures within complex, multimodality datasets to generate hypotheses that may not have been evident previously. We have demonstrated this method with a pilot portion of musculoskeletal data from the ADVANCE study and generated a novel hypothesis regarding head injury.

## Supplementary material

10.2196/91958 Multimedia Appendix 1 Supplementary files containing all the latent variables and matched loadings identified in sparse group factor analysis.

## Abbreviations

ADVANCE: The Armed Services Trauma and Rehabilitation Outcome
AI: artificial intelligence
DEXA: dual-energy X-ray absorptiometry
EQ-5D-5L: EuroQoL 5-Dimensions 5-Level
GFA: group factor analysis
JTTR: Joint Theatre Trauma Registry
NISS: New Injury Severity Score
TBI: traumatic brain injury
