# Supplementary material for: Novel Musculoskeletal Hypotheses in the Armed Services Trauma and Rehabilitation Outcome (ADVANCE) Cohort: Development and Application of Sparse Group Factor Analysis Methodology
Source: J Med Internet Res. 2026 Jul 21;28:e91958. doi: 10.2196/91958 (PMC13386667; doi:10.2196/91958)

# Supplementary files

S1: All latent variables identified by sparse Group Factor Analysis.


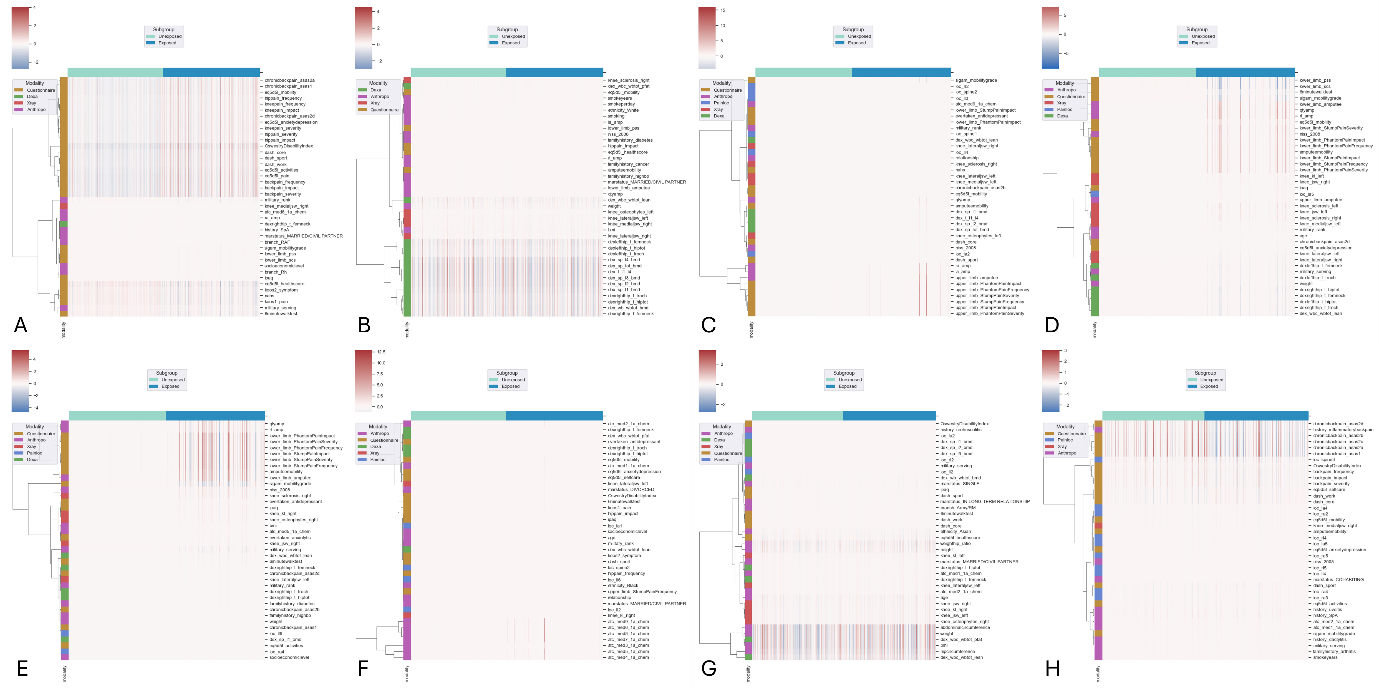


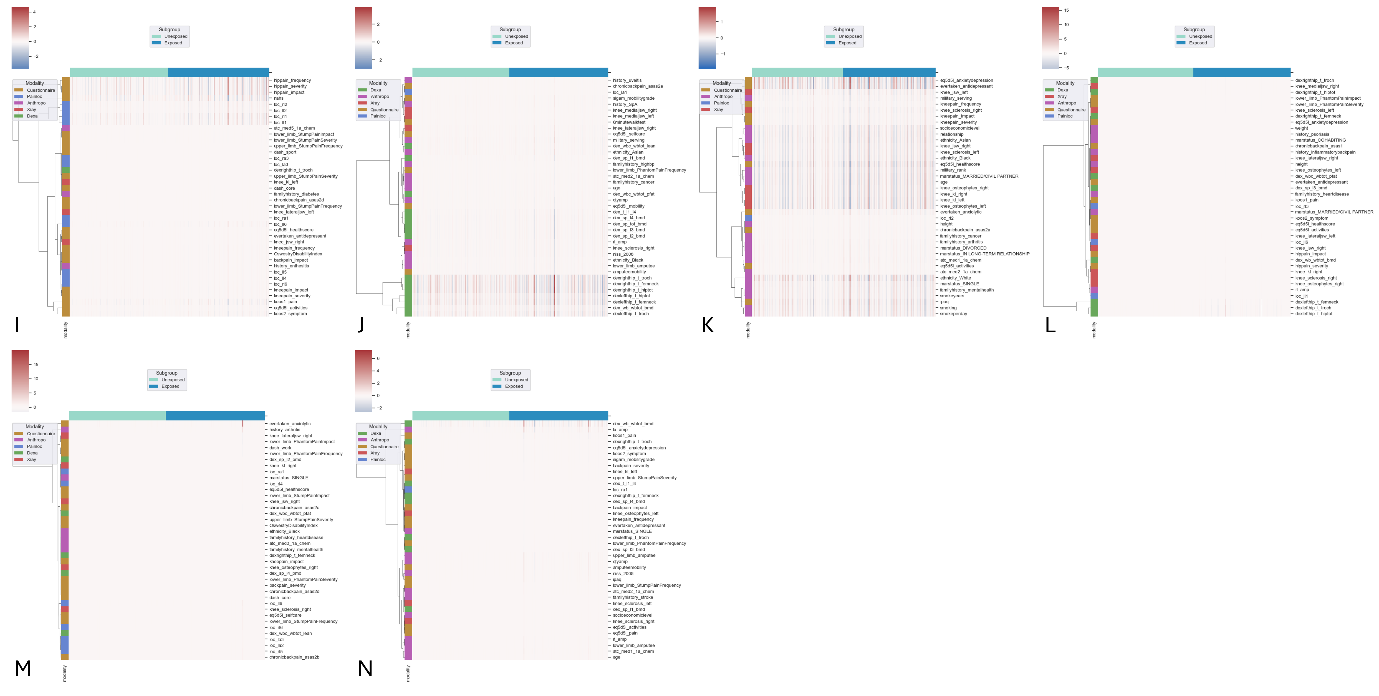


S2: Matched loadings of all latent variables identified by sparse Group Factor Analysis.


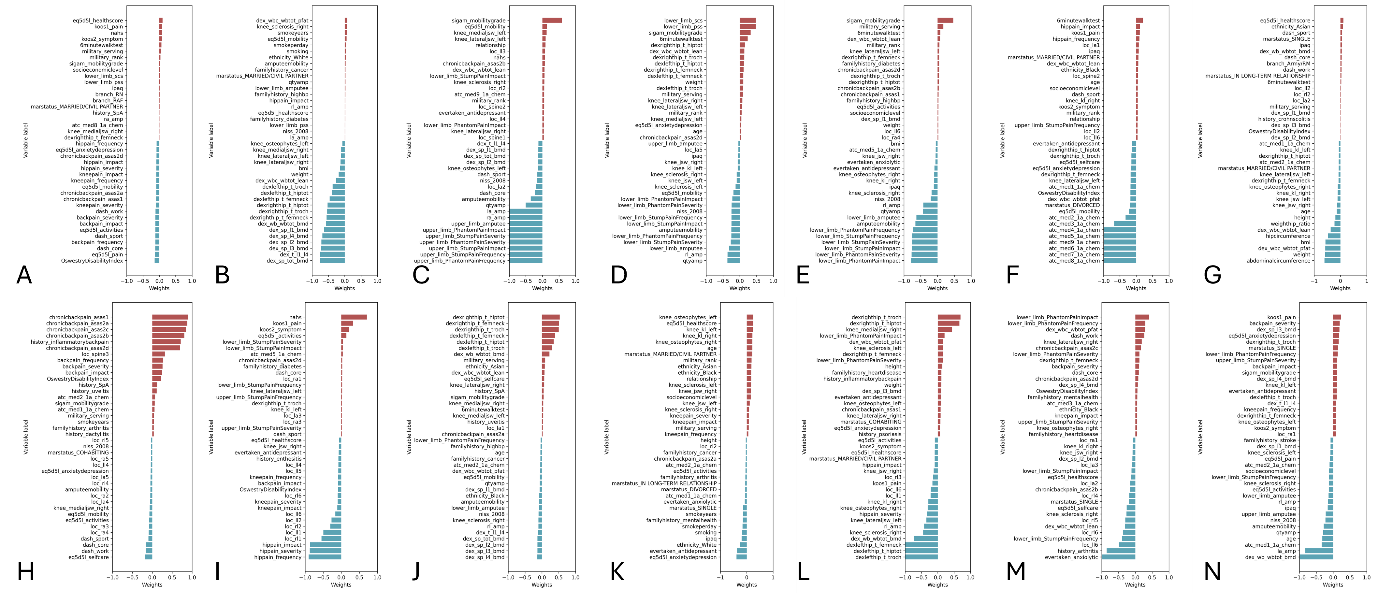

Supplement: Multimedia Appendix 1 [file jmir-v28-e91958-s001.docx]
